# Supplementary material for: A Metabolomics Analysis of Postmenopausal Breast Cancer Risk in the Cancer Prevention Study II
Source: Metabolites. 2021 Feb 10;11(2):95. doi: 10.3390/metabo11020095 (PMC7916573; doi:10.3390/metabo11020095)
Supplement: Supplementary file 1 [file metabolites-11-00095-s001.zip › Supplementary Tables CPSII metabolomics breast cancer 3.0.docx]

**Supplementary Table 1.** Odds ratios, 95% confidence intervals, p-values, and q-values for postmenopausal breast cancer when comparing the 90th with the 10th percentile levels of metabolites in conditional logistic models adjusted for age at blood draw (please refer to Supplementary_Table_1.xlsx).

**Supplementary Table 2**. Age-adjusted odds ratios (ORs) and 95% confidence intervals (CIs) among non-hormone users at baseline (N=319) for postmenopausal breast cancer when comparing the 90^th^ with the 10^th^ percentile levels of metabolites (top 20 metabolites by statistical significance)

| Metabolite | OR (95% CI) | P-value | Q-value |
| --- | --- | --- | --- |
| X – 11795 | 2.58 (1.70-3.90) | 7.34E-06 | 0.01 |
| X – 24293 | 2.22 (1.45-3.40) | 0.0003 | 0.11 |
| 1-palmitoyl-2-palmitoleoyl-GPC (16:0/16:1) | 2.18 (1.40-3.39) | 0.0006 | 0.18 |
| Androstenediol (3beta,17beta) monosulfate (2) | 1.93 (1.32-2.82) | 0.0007 | 0.18 |
| 1-palmitoyl-2-oleoyl-GPC (16:0/18:1) | 1.93 (1.27-2.91) | 0.0019 | 0.34 |
| Androstenediol (3beta,17beta) disulfate (1) | 1.90 (1.29-2.81) | 0.0012 | 0.26 |
| Androsteroid monosulfate (1) | 1.83 (1.21-2.75) | 0.0039 | 0.42 |
| 2-palmitoleoyl-GPC (16:1) | 1.82 (1.18-2.79) | 0.0065 | 0.41 |
| Androstenediol (3beta,17beta) monosulfate (1) | 1.75 (1.17-2.60) | 0.0061 | 0.43 |
| X – 18240 | 1.64 (1.17-2.31) | 0.0046 | 0.42 |
| Ethyl glucuronide | 1.63 (1.17-2.29) | 0.0044 | 0.43 |
| 5alpha-androstan-3alpha,17beta-diol monosulfate (2) | 1.35 (1.10-1.66) | 0.0039 | 0.49 |
| Sphingosine 1-phosphate | 0.64 (0.46-0.88) | 0.0055 | 0.41 |
| 1-(1-enyl-oleoyl)-GPE (P-18:1) | 0.59 (0.41-0.84) | 0.0039 | 0.46 |
| X – 18901 | 0.58 (0.40-0.85) | 0.0047 | 0.40 |
| X – 13729 | 0.58 (0.40-0.86) | 0.0063 | 0.42 |
| 1-(1-enyl-stearoyl)-GPE (P-18:0) | 0.57 (0.39-0.83) | 0.0037 | 0.52 |
| X – 13866 | 0.57 (0.39-0.85) | 0.0051 | 0.41 |
| X – 16343 | 0.51 (0.33-0.78) | 0.0020 | 0.32 |
| 4-allylphenol sulfate | 0.45 (0.30-0.67) | 7.74E-05 | 0.05 |

**Supplementary Table 3**. Age-adjusted odds ratios (ORs) and 95% confidence intervals (CIs) for estrogen receptor positive breast cancer (N=644) when comparing the 90^th^ with the 10^th^ percentile levels of metabolites (top 20 metabolites by statistical significance)

| Metabolite | OR (95% CI) | P-value | Q-value |
| --- | --- | --- | --- |
| X – 21470 | 1.77 (1.23-2.55) | 0.002 | 0.26 |
| X – 24293 | 1.73 (1.24-2.41) | 0.001 | 0.23 |
| Androsteroid monosulfate (1) | 1.68 (1.24-2.27) | 0.0008 | 0.23 |
| Androstenediol (3beta,17beta) monosulfate (2) | 1.66 (1.25-2.20) | 0.0004 | 0.23 |
| X – 12112 | 1.62 (1.17-2.26) | 0.004 | 0.31 |
| Androstenediol (3beta,17beta) disulfate (1) | 1.61 (1.22-2.14) | 0.0009 | 0.23 |
| 16alpha-hydroxy DHEA 3-sulfate | 1.60 (1.21-2.13) | 0.001 | 0.23 |
| Androstenediol (3beta,17beta) monosulfate (1) | 1.55 (1.16-2.07) | 0.003 | 0.30 |
| X – 24546 | 1.55 (1.15-2.07) | 0.004 | 0.31 |
| 5alpha-androstan-3beta,17beta-diol disulfate | 1.51 (1.13-2.00) | 0.005 | 0.31 |
| Threonate | 0.66 (0.50-0.88) | 0.004 | 0.31 |
| X – 16944 | 0.66 (0.50-0.88) | 0.004 | 0.31 |
| X – 21319 | 0.66 (0.49-0.88) | 0.004 | 0.31 |
| X – 18921 | 0.65 (0.49-0.88) | 0.005 | 0.31 |
| X – 18603 | 0.65 (0.50-0.85) | 0.001 | 0.26 |
| X – 12015 | 0.63 (0.46-0.86) | 0.004 | 0.31 |
| Linolenoylcarnitine (C18:3) | 0.63 (0.47-0.84) | 0.001 | 0.26 |
| 1-palmitoleoyl-2-linolenoyl-GPC (16:1/18:3) | 0.58 (0.41-0.82) | 0.002 | 0.26 |
| 4-allylphenol sulfate | 0.58 (0.44-0.78) | 0.0002 | 0.23 |
| 3,4-methyleneheptanoylcarnitine | 0.56 (0.40-0.79) | 0.001 | 0.23 |
